# Radiopacity Evaluation of Contemporary Luting Cements by Digitization of Images

**DOI:** 10.5402/2012/704246

**Published:** 2012-09-13

**Authors:** José Maurício dos Santos Nunes Reis, Érica Gouveia Jorge, João Gustavo Rabelo Ribeiro, Ligia Antunes Pereira Pinelli, Filipe de Oliveira Abi-Rached, Mário Tanomaru-Filho

**Affiliations:** ^1^Department of Dental Materials and Prosthodontics, Araraquara Dental School, São Paulo State University (UNESP), 14801-903 Araraquara, SP, Brazil; ^2^Department of Restorative Dentistry, Araraquara Dental School, São Paulo State University (UNESP), 14801-903 Araraquara, SP, Brazil; ^3^Department of Restorative Dentistry, Dental School of Três Corações, Vale do Rio Verde University (UNINCOR), 37410-000 Três Corações, MG, Brazil

## Abstract

*Objective*. The aim of this study was to evaluate the radiopacity of two conventional cements (Zinc Cement and Ketac Cem Easymix), one resin-modified glass ionomer cement (RelyX Luting 2) and six resin cements (Multilink, Bistite II DC, RelyX ARC, Fill Magic Dual Cement, Enforce and Panavia F) by digitization of images. *Methods*. Five disc-shaped specimens (10 × 1.0 mm) were made for each material, according to ISO 4049. After setting of the cements, radiographs were made using occlusal films and a graduated aluminum stepwedge varying from 1.0 to 16 mm in thickness. The radiographs were digitized, and the radiopacity of the cements was compared with the aluminum stepwedge using the software VIXWIN-2000. Data (mmAl) were submitted to one-way ANOVA and Tukey's test (*α* = 0.05). *Results*. The Zinc Cement was the most radiopaque material tested (*P* < 0.05). The resin cements presented higher radiopacity (*P* < 0.05) than the conventional (Ketac Cem Easymix) or resin-modified glass ionomer (RelyX Luting 2) cements, except for the Fill Magic Dual Cement and Enforce. The Multilink presented the highest radiopacity (*P* < 0.05) among the resin cements. *Conclusion*. The glass ionomer-based cements (Ketac Cem Easymix and RelyX Luting 2) and the resin cements (Fill Magic Dual Cement and Enforce) showed lower radiopacity values than the minimum recommended by the ISO standard.

## 1. Introduction

 Cementation of fixed partial dentures to dental implant abutments or tooth preparations is among the clinical applications for dental luting cements. One desirable property that cements should present is enough radiopacity [1, 2], which allows to detect excess cement into the soft tissues around tooth- [3] and implant-supported prostheses [4], easing the removal of cement overhangs. This aspect is important mainly for cement retained implant prostheses when subgingival margins are present, considering the adverse effects of extruded cement for the periimplant soft tissues [4]. According to Weber et al. [5], the cement retained restorations presented poorer soft tissue health in relation to screw retained ones. 

Moreover, the radiopacity of the luting agents is critical in the diagnosis of persistent decay and assessment of open gingival margins [1, 6]. Therefore, it is important that the cement presents greater radiopacity than dentin [3]. On the other hand, when considering implants, titanium or other metals must be taken into account, once the luting agent should be more radiopaque than those materials [7]. Eliasson and Haasken [8] established a comparison standard for radiopacity studies, using optical radiographic density measurements for impression materials and an equivalent thickness of aluminum capable of producing similar radiographic density. More recently, Tanomaru-Filho et al. [9] evaluated the radiopacity by digitization of images of five root canal sealers using a graduated aluminum stepwedge varying from 2.0 to 16 mm in thickness. The authors concluded that the use of digitized images and computer-aided radiographic image analysis with computer programs allowed the development of radiopacity studies that are simply executed, reproducible, and able to provide reliable results. Other studies [7, 10] have evaluated the radiopacity of dental materials using an aluminum stepwedge as a comparison standard.

The use of resin luting cements has increased considerably over the last few years [11]. Some resin and resin-modified glass ionomer cements have been developed with improved physical properties when compared with glass ionomer or zinc phosphate [6], being the latter the most widely used dental luting cement for decades [12, 13]. Resin cements are available in autopolymerization, light-polymerization, and dual-polymerization formulations [14]. The chemical composition, the amounts of fillers and components of the organic matrix, and the atomic weight of the filler particles may influence the radiopacity of these materials [15, 16]. 

Considering the increasing use of resin-based luting materials for both tooth- or implant-supported restorations, it is important to evaluate their physical and chemical properties, including radiopacity. With this in mind, the aim of this in vitro study was to evaluate the radiopacity of nine dental luting cements by digitization of images. The null hypothesis tested was that the material type would not affect the radiopacity of dental luting cements.

## 2. Materials and Methods

 Nine luting cements were evaluated in this study (Table 1). Zinc Cement was used as the control group. The materials were manipulated according to manufacturers' instructions, and the tests were carried out at room temperature (23 ± 1°C). Standardized specimen discs were produced using circular stainless steel patterns with 10 mm diameter and 1.0 mm thickness. Molds of these metallic patterns were taken using a light-bodied silicone-based impression material and then placed on a 0.5 mm clean glass slide. The cements were mixed, inserted into the silicone molds, and, to allow overflow of excess material, a second glass slide was positioned on top of the filled silicone molds, which remained sandwiched until setting of the luting cements. The dual-polymerizing resin cements were photopolymerized for 40 s, using a halogen light source (XL 3000, 3 M ESPE, St. Paul, MN, USA) with an intensity of 600 mW/cm^2^. Five specimens, measuring 10 mm diameter by 1.0 mm thickness, were made for each tested material. The thickness of each specimen was verified with a digital caliper at three locations to within 0.01 mm tolerance. Thereafter, the specimens were stored at 37°C for 24 h, before the X-rays sets.

**Figure 1:**
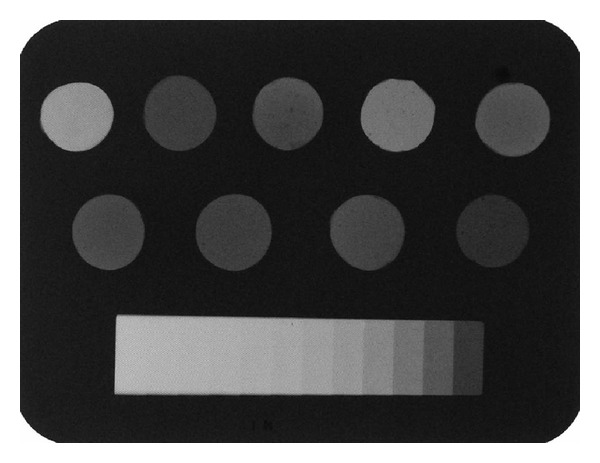
Radiographic film obtained with one disc of each material and the graduated aluminum stepwedge.

 After storage, the specimens were positioned on five occlusal radiographic films (Insight-Kodak Comp, Rochester, NY, USA) and exposed, along with an aluminum stepwedge with variable thickness (from 1.0 to 16.0 mm, in 1.0 ± 0.01 mm increments per step). A GE-1000 X-ray unit (General Electric, Milwaukee, WI, USA) operating at 50 kvp, 10 mA, 18 pulses/s, and focus-film distance of 33.5 cm was used [9]. The films were processed in a standard automatic processor (Dent-X 9000, Dent-X, Elmsford, USA). Radiographs were digitized (Figure 1) using a desktop scanner (SnapScan 1236-Agfa, Deutschland) with 600 dpi resolution. The images were saved as uncompressed TIFF files and imported into the VIXWIN-2000 software (Gendex, Des Plaines, IL, USA), where an equal-density tool was used to identify equal-density areas in the images [9]. This software shows the radiograph images at various magnifications and the tonal range of every point on a gray level scale of 1 to 255 pixels. The mouse-driven probe of the program assesses continuously the gray level in pixels at any point of the images on the screen. The value for the sample was measured at selected points after surveying the entire surface of the specimen searching for homogenous regions and avoiding those that are obviously not typical, such as areas containing entrapped air bubbles. Functions that change key parameters, such as luminosity, contrast, and equalization were not used to avoid introducing artifacts that could bias the results. This procedure allowed comparison between the radiographic densities of the various cements and the radiopacity of different degrees of thickness of the aluminum stepwedge detected by the equal density tool.

Results were analyzed by calculating the means of five measurements per sample (one point in the central area and four points in the different quadrants) [9]. Data (mm Al) were submitted to 1-way analysis of variance (ANOVA) and Tukey's HSD post hoc test (*α* = 0.05).

## 3. Results

**Figure 2:**
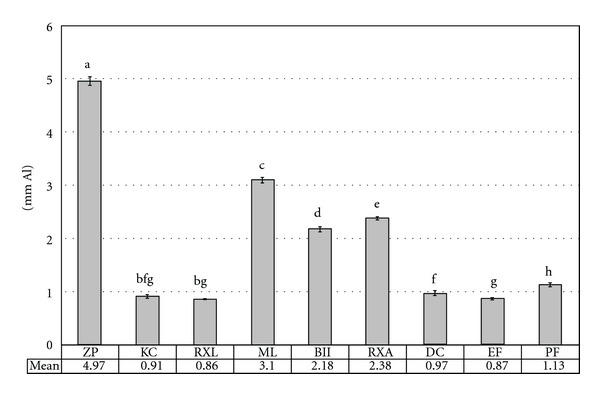
Mean radiopacity values and standard deviation (vertical bars) of the materials, and the results of Tukey's HSD post hoc test. Columns with the same letter were not statistically different (*P* > 0.05).

Statistical analysis showed a significant difference among the mean radiopacity values of the luting cements tested. Mean values, standard deviation, and Tukey's post hoc test results are presented in Figure 2. The Zinc Cement presented the highest radiopacity (*P* < 0.05) among the tested materials. No significant difference was found neither between Ketac Cem Easymix, RelyX Luting 2, and Enforce (*P* ≥ 0.05) nor between Ketac Cem Easymix and Fill Magic Dual Cement (*P* ≥ 0.05). With the exception of Fill Magic Dual Cement and Enforce, the resin cements presented higher (*P* < 0.05) radiopacity than the glass ionomer-based cements. Among the resin cements, Multilink presented the highest mean radiopacity value (*P* < 0.05), followed by RelyX ARC, Bistite II DC, and Panavia F, which presented different results among them (*P* < 0.05).

## 4. Discussion

 Luting cements are a kind of material used to attach and seal metallic and aesthetic posts, dental restorations, and prostheses to teeth [14, 17] and also to retain implant restorations [7]. According to Attar et al. [6], the choice of a dental luting cement is dependent on the clinical situation combined with the physical, biologic, and handling properties of the material. One factor that must be considered is that the radiopacity of the dental luting cements is critical in the diagnosis of recurrent decay, in the detection of open gingival margins and residual material. In addition, when a luting agent has lower radiopacity than dentin, it is difficult to radiographically detect a cement line of post or restorative crowns [18]. 

The radiopacity property should also be considered when selecting cement for implants restorations, with the aim of determining the presence of excess cement and confirming the correct positioning of cemented units, mainly when implant-abutment interface is located subgingivally [7]. According to Wilson Jr. [4], cement overhangs are associated with signs of peri-implant disease. Therefore, studies of basic properties, physical and mechanical, including radiopacity, are necessary to characterize newer materials in relation to the more traditional cements.

The ISO 4049/2000 [19] establishes that the radiopacity of the materials should be equal to or greater than that of the same thickness of aluminum. In addition, according to Gu et al. [2], of all the ISO and ANSI/ADA requirements for dental materials, the lowest radiopacity requirement is 1.0 mm of aluminum per mm of the material. Within the results of the present study, with the exception of the glass ionomer-based cements (Ketac Cem Easymix and RelyX Luting 2) and two resin cements (Fill Magic Dual Cement and Enforce), all materials had radiopacity values above the minimum recommended by the ISO 4049/2000 [19]. 

 The physical properties of dental luting cements could vary considerably because of differences in the quantity and quality of their chemical components [20, 21]. In the present study, the Zinc Cement was the most radiopaque material. Similar outcomes were found by several authors, when this type of cement was studied [1, 6, 7]. The resin cements presented higher radiopacity than the conventional or resin-modified glass ionomer cements, except for Fill Magic Dual Cement and Enforce. According to Watts [15], the inclusion of elements with high atomic weight in the filler particles of resin-based luting cements contributes to an increased radiopacity. Several types of inorganic fillers may be responsible for the difference on the radiopacity of the materials [1, 6]. According to Attar et al. [6], the filler particles that provide radiopacity to zinc phosphate, glass ionomer, and resin luting cements are zinc oxide, magnesium oxide, fluoroaluminosilicate glass, barium, strontium, and zirconium. 

Zinc Cement contains zinc oxide, magnesium oxide and aluminum hydroxide which contributes to its greater radiopacity. Wadhwani et al. [7] comment that the glass ionomers and resin cements are expected to have poor radiodensity properties unless specific radiopacifiers are added during formulation. According to the manufacturer, Multilink resin cement contains ytterbium trifluoride, a good radiopacifier and fluoride releaser agent [22, 23]. As previously commented, this autopolymerizing material presented the highest radiopacity among the resin cements. RelyX ARC contains approximately 67.5% by weight of the zirconia/silica filler as purported by the manufacturer. The high percentage of this compound can be associated to its radiopacity [24]. Bistite II DC also contains a high percentage of zirconia/silica filler (77 wt%), acting as the radiopacifier. The manufacturer of Panavia F does not inform the presence of a specific radiopacifier agent. Therefore, the radiopacity of this cement is probably associated to the silica fillers present in its chemical composition. With respect to Fill Magic Dual Cement, the manufacturer does not report its radiopacifier agent, and its chemical composition is based on metacrylic monomer, silica, radiopaque filler, and fluor. The Enforce resin cement contains barium-aluminum glass and titanium dioxide as radiopacifier agents, while the RelyX luting 2 resin-modified glass ionomer cement contains a radiopaque fluoroaluminosilicate glass and nonreactive zirconia silica filler acting as the radiopacifier. Finally, Ketac Cem Easymix contains only fluoroaluminosilicate glass, a typical chemical component of conventional glass-ionomer cements, as radiopacifier agent. The lower results of radiopacity of the glass-ionomer cements evaluated in this study are consistent with those of previous studies [7, 9]. Williams and Billington [25] reported that the incorporation of alumino silicate glass alone favors the translucency of the glass ionomer materials. Future studies evaluating the infrared spectra of these materials should include reliable information about the agents used as radiopacifiers. 

It is important to observe that oral environment conditions were not simulated in this investigation, which could influence the results by moisture adsorption. This study evaluated the radiopacity of dental luting cements under in vitro conditions, as recommended by ISO standard [19]. Advancement in estimating the clinical radiopacity of the materials under indirect tooth or implant restorations should be considered in further investigations.

## 5. Conclusion

According to the methodology of the present study it may be concluded that the luting cements evaluated had different radiopacities. The glass ionomer-based cements (Ketac Cem Easymix and RelyX Luting 2) and two resin cements (Fill Magic Dual cement and Enforce) had radiopacity values below the minimum recommended by the ISO standard. Therefore, these materials should be carefully used in situations where the cement/restoration margin is located in a difficult access area to remove the excess cement and verify the correct marginal adaptation, favoring adverse effects to periodontal-implant soft tissues and recurrent decay. 

## Figures and Tables

**Table 1:** Luting cements tested.

Product	Code	Type	Manufacturer
Zinc Cement	ZP	Zinc phosphate	S.S. White Ltda., Rio de Janeiro, RJ, Brazil
Ketac Cem Easymix	KC	Glass ionomer	3M ESPE, St. Paul, MN, USA
RelyX Luting 2	RXL	Self-curing resin-modified glass ionomer	3M ESPE, St. Paul, MN, USA
Multilink	ML	Autopolymerizing resin	Ivoclar-Vivadent, Schaan, Liechtenstein, Germany
Bistite II DC	BII	Dual-polymerizing resin	Tokuyama Dental Corporation, Taitou-Ku, Tokyo, Japan
RelyX ARC	RXA	Dual-polymerizing resin	3M ESPE, St. Paul, MN, USA
Fill Magic Dual Cement	DC	Dual-polymerizing resin	Vigodent S/A, Rio de Janeiro, RJ, Brazil
Enforce	EF	Dual-polymerizing resin	Dentsply Indústria e Comércio Ltda., Petrópolis, RJ, Brazil
Panavia F	PF	Dual-polymerizing resin	Kuraray Medical Inc., Kurashiki, Okayama, Japan
